# Neuromodulation for Cannabis Use: A Scoping Review

**DOI:** 10.3390/brainsci14040356

**Published:** 2024-04-02

**Authors:** Anthony Ngoy, Victor M. Tang, Kebin Xiao, Daniel M. Blumberger, Tony P. George, Joshua L. Gowin, Bernard Le Foll, Matthew E. Sloan

**Affiliations:** 1 Addictions Division, Centre for Addiction and Mental Health, Toronto, ON M6J 1H4, Canada; anthony.ngoy@camh.ca (A.N.); tony.george@camh.ca (T.P.G.); bernard.lefoll@camh.ca (B.L.F.); 2 Department of Pharmacology & Toxicology, University of Toronto, Toronto, ON M5G 2C8, Canada; 3 Temerty Centre for Therapeutic Brain Intervention, Centre for Addiction and Mental Health, Toronto, ON M6J 1H4, Canada; victor.tang@camh.ca (V.M.T.); daniel.blumberger@camh.ca (D.M.B.); 4 Department of Psychiatry, University of Toronto, Toronto, ON M5T 1R8, Canada; 5 Campbell Family Mental Health Research Institute, Centre for Addiction and Mental Health, Toronto, ON M6J 1H4, Canada; 6 Institute of Medical Science, University of Toronto, Toronto, ON M5S 1A8, Canada; 7 Institute for Mental Health Policy Research, Centre for Addiction and Mental Health (CAMH), Toronto, ON M5S 2S1, Canada; 8 Departments of Radiology and Psychiatry, University of Colorado School of Medicine, Aurora, CO 80045, USA; joshua.gowin@cuanschutz.edu; 9 Translational Addiction Research Laboratory, Centre for Addiction and Mental Health (CAMH), Toronto, ON M5S 2S1, Canada; 10 Department of Family and Community Medicine, Faculty of Medicine, University of Toronto, Toronto, ON M5G 1V7, Canada; 11 Waypoint Research Institute, Waypoint Centre for Mental Health Care, Penetanguishene, ON L9M 1G3, Canada; 12 Department of Psychological Clinical Science, University of Toronto Scarborough, Toronto, ON M1C 1A4, Canada

**Keywords:** cannabis use, neuromodulation, transcranial magnetic stimulation, transcranial direct current stimulation, deep brain stimulation

## Abstract

This scoping review explores the use of neuromodulation techniques in individuals with cannabis use. Our goal was to determine whether cannabis use alters cortical excitation and inhibition in the context of neuromodulation and to determine whether neuromodulation affects craving and cannabis use patterns. A systematic search was conducted using PubMed, OVID Medline, and PsycINFO from inception to 20 December 2022. Our review identified ten relevant studies, eight of which used Transcranial Magnetic Stimulation (TMS), while two employed Transcranial Direct Current Stimulation (tDCS). Findings from TMS studies suggest that cannabis users exhibit altered cortical inhibition, with decreased short interval intracortical inhibition (SICI) compared to non-users. Single sessions of rTMS did not have any impact on cannabis craving. By contrast, two studies found that multiple sessions of rTMS reduced cannabis use, but these changes did not meet the threshold for statistical significance and both studies were limited by small sample sizes. The two included tDCS studies found contradictory results, with one showing reduced cannabis craving with active treatment and another showing no effect of active treatment on craving compared to sham. Future studies should further explore the effects of multiple treatment sessions and different neuromodulation modalities.

## 1. Introduction

According to the United Nations Office on Drugs and Crime, cannabis is the most widely used illicit drug globally, with an estimated 209 million users in 2020 [1]. In addition to recreational use, many people use cannabis for medical reasons, with chronic pain being one of the most commonly cited conditions. Enrollment in medical cannabis programs in the U.S. has increased dramatically in recent years, although this appears to be driven by states where recreational use has not been legalized [2]. Legalization of cannabis use has allowed adults to grow, possess, and purchase cannabis for personal use in some countries and U.S. states, allowing cannabis sales to be taxed and regulated, which has important economic implications. For example, in Canada, annual sales of cannabis for non-medical purposes totaled $4.5 billion in 2022 [3]. Legalization has also had criminal justice system ramifications, leading to significant decreases in cannabis-related charges or arrests in Canada [4] and the U.S. [5]. However, the increased acceptance of cannabis use has led to greater prevalence of some hazardous behaviors. For example, the frequency of motor vehicle accidents in British Columbia involving drivers under the influence of cannabis has increased from 9.2% to 17.9% since the legalization of cannabis in Canada [6]. Studies have found that commercialization of cannabis, as opposed to legalization, led to higher rates of emergency department visits for cannabis-induced psychosis [7] and cannabis hyperemesis syndrome [8] in Ontario. In addition to these sequelae of cannabis use, approximately 10% of users will go on to develop cannabis use disorder (CUD) [9]. Given the high prevalence of cannabis use and important health consequences of heavy use, identifying treatments for problematic cannabis use is necessary to reduce the global health burden associated with cannabis consumption.

Cannabis use disorder is a condition in which individuals have difficulty controlling their cannabis intake despite negative consequences of use. Globally, CUD is estimated to affect about 22 million people [10]. There are no approved pharmacotherapies for CUD [11], although some newer medications show promise, such as FAAH inhibitors [12], the cannabinoid receptor 1 (CB1) signaling specific inhibitor AEF0117 [13], and CB1 agonists, such as nabixomols [14]. Psychotherapies, such as cognitive-behavioral therapy (CBT) and motivational enhancement therapy (MET), reduce cannabis use in the short-term, although the effects of treatment tend to wane over time [15]. Contingency management for cannabis addiction has also been shown to be effective at reducing use and increasing abstinence rates on its own or in combination with CBT and MET, including for those diagnosed with comorbid psychotic or depressive disorders [16]. Although these interventions have short-term efficacy, treatment options for CUD are limited, and many individuals do not benefit from existing treatments.

A novel approach to treating substances use disorders is neuromodulation. These modalities represent a variety of techniques using electrical or electromagnetic stimulation to targeted brain regions, with the goal of correcting the function of neurocircuitry implicated in these disorders. Transcranial magnetic stimulation (TMS) is a neuromodulation technique using electromagnetic induction by delivering electrical current through a coil that is placed over the scalp, generating a focal electric field in the superficial cortex below [17]. When TMS is applied in a series of pulses, called a train, it is known as repetitive transcranial magnetic stimulation (rTMS). The motor threshold (MT) is the amount of energy required to elicit movement of the thumb on the contralateral side of the body, which helps determine the dose of stimulation of treatment [17]. With multiple trains of rTMS, another important factor is the frequency of pulsing, which can be further categorized into low frequency (<1 Hz), which is inhibitory, or high frequency (>5 Hz), which is excitatory. Typically, figure-eight coils are used [17]. The dorsolateral prefrontal cortex (DLPFC) has been an area of focus in substance use disorders due to its involvement with regulation of the reward system and executive functioning [18]. When rTMS is applied to the DLPFC, it has been found to reduce cravings compared to sham in those with alcohol and nicotine addiction [19]. Based on two positive trials, Health Canada and the Food and Drug Administration (FDA) recently cleared an rTMS treatment that targets both the DLPFC and the insula for smoking cessation [20,21]. Other neuromodulation techniques such as deep brain stimulation (DBS), which uses surgically implanted electrodes to deliver electric stimulation, and transcranial direct current stimulation (tDCS), which uses low electrical currents via electrodes on the scalp, have been explored for the treatment of substance use disorders [22]. Preliminary evidence suggests that deep brain stimulation of the nucleus accumbens is effective for treatment-resistant alcohol use disorder with increased abstinence days and decreased cravings [23]. If similar treatments are effective for CUD, they could expand the range of treatment options.

Brain stimulation alters neuronal activity and can be used to excite or inhibit different brain regions. Excitation refers to the process of increasing neural activity within the cortex, leading to the generation and transmission of electrical impulses, whereas cortical inhibition involves the suppression or reduction of neural activity within the cortex [24]. TMS coils can generate a magnetic field in brain tissue that creates an electric current able to depolarize neurons. A single TMS pulse over the motor cortex can induce a motor-evoked potential (MEP) that can measured with electromyography (TMS-EMG) [25], or when a pulse is delivered over non-motor regions such as the frontal cortex, cortical TMS evoked potentials can be measured with electroencephalography (TMS-EEG) to measure other areas of the brain where this activity propagates [26]. Furthermore, several single- and paired-pulse TMS-EEG and TMS-EMG paradigms have been developed to index measures of cortical inhibition and neuroplasticity [27], effectively providing a novel method for probing mechanisms of disease and biomarkers of treatment response with the use of neuromodulation techniques. There are several measures that can be used to assess cortical inhibition and excitation: short-interval intracortical inhibition (SICI), long-interval intracortical inhibition (LICI), intracortical facilitation (ICF), and cortical silent period (CSP). SICI, ICF, and LICI are paired-pulse TMS techniques. By applying a test stimulus following a conditioning stimulus, different test responses will occur based on the interstimulus intervals. Specifically, SICI is measured by applying a test stimulus shortly after a conditioning stimulus (1–4 ms), ICF is measured when the test stimulus is 10–15 ms after the conditioned stimulus, and LICI is measured when the test stimulus is performed 100 ms or more after the conditioned stimulus [28]. SICI and LICI are thought to reflect activity at GABA receptors. CSP is the transient period of inhibition following suprathreshold TMS-induced MEP [29]. In the previous literature, these measures have been reported to differ in individuals with psychiatric and neurological disorders compared to healthy individuals [27]. Understanding how cortical activity is altered in cannabis users via neuromodulation can potentially provide useful information about how cannabis use alters brain function.

**Figure 1:**
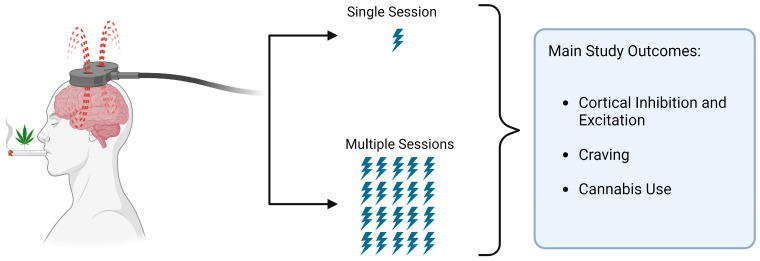
Main outcomes for included neuromodulation studies.

This scoping review aims to summarize the existing literature on the use of neuromodulation therapies in understanding the pathophysiology and treatment of problematic cannabis use. We have two main aims: (1) to determine whether cannabis use alters cortical excitation and inhibition in the context of neuromodulation and (2) to determine whether neuromodulation affects cannabis use and craving in heavy cannabis users (Figure 1). We will summarize the study design and findings of neuromodulation studies for heavy cannabis use. Further, we aim to delineate gaps in the literature and recommend next steps for neuromodulation studies targeting individuals with cannabis use disorder.

## 2. Materials and Methods

Our review follows the PRISMA guidelines for scoping reviews [30]. A literature search was conducted through the electronic databases PubMed, OVID Medline, and PsycInfo on 20 December 2022. Studies were identified using a search employing terms related to cannabis use and terms relating to brain stimulations therapies. Terms that were used include: ‘cannabis use’, ‘marijuana use’, ‘brain stimulation’, ‘neuromodulation’, ‘neurostimulation’, ‘Deep Brain Stimulation’, ‘Transcranial magnetic stimulation’, ‘electroconvulsive therapy’, ‘Transcranial Direct Current Stimulation’, ‘Nerve Stimulation’, ‘DBS’, ‘rTMS’, ‘ECT’, and ‘tDCS’ (Supplementary Tables S1–S3).

We included human studies in which neuromodulation was administered in the context of understanding or treating problematic cannabis use. To be included, participants needed to be 18 years of age or older and be diagnosed with CUD using any diagnostic tool or assessment (e.g., SCID-5, MINI) or using cannabis heavily. Interventions included any form of neuromodulation (i.e., TMS, rTMS, tDCS). Outcomes were measures of cortical excitability and inhibition, cannabis use, and craving. The full text of the article needed to be available in English. Only original studies were considered; review papers were excluded.

The papers were selected by two independent reviewers based on the eligibility criteria using the web-based software, Covidence (Veritas Health Innovation) (AN & KX). Duplicate papers were removed if uploaded from multiple databases to Covidence. Abstracts were screened and relevant papers underwent full-text review. Any discrepancies in the study selection were discussed at full-text review and resolved before moving onto the next stage of selection (MES).

Data extracted from the selected papers included first author, year of publication, study objective, study design, study type, location of study, year, inclusion/exclusion criteria, sample size, sample user type, age, sex, cannabis use assessment, neuromodulation type, target area, number of sessions, intensity, frequency, measures, and main findings (AN).

**Figure 2:**
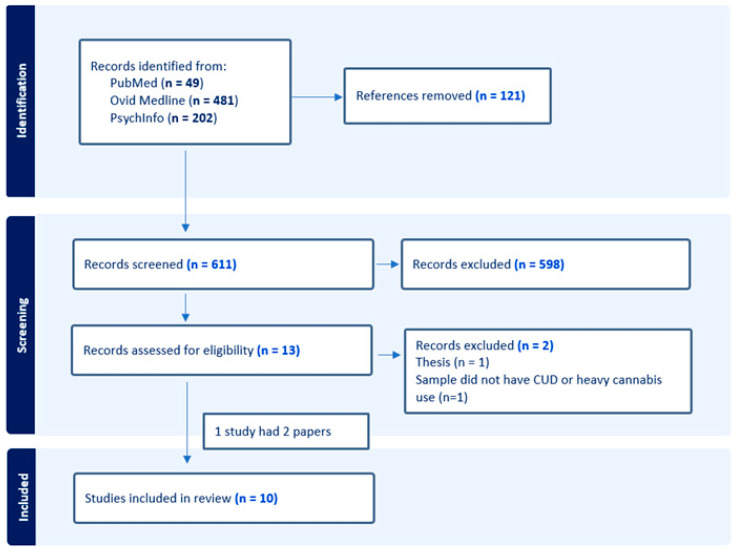
PRISMA flow diagram.

There were 732 papers that were identified using the literature search; these were screened by their titles and abstracts. Out of these, 13 were eligible for full-text review. Twelve of the articles assessed by full-text review were included. One paper was excluded as it was a thesis that was subsequently published as a peer-revised paper which was included in our review. One study was excluded, as the sample did not meet criteria of CUD or heavy cannabis use. A summary of the search process is shown below in Figure 2.

## 3. Results

### 3.1. Study Selection

Ten studies were included in the analysis. Of these studies, eight utilized TMS [24,31,32,33,34,35,36,37,38] and two utilized tDCS [39,40]. No studies used other forms of neuromodulation. Four studies investigated cortical inhibition and excitability of cannabis users [24,34,35,38], whereas the other six studies examined the effects of brain stimulation on clinical outcomes such as craving and/or cannabis use behavior [31,32,33,36,37,39,40]. One of the studies had two papers [33,36] reporting different results from the same trial.

### 3.2. Study Characteristics

Samples typically consisted of young adult cannabis users; the mean age of the sample was below 40 for all included studies (Table 1). Studies had predominantly male samples, with three of the ten studies having at least one participant group with no females [24,33,35,36]. Sample sizes of included studies ranged from 16 to 62. Studies described cannabis use in several different ways, including joint-years [33,36], grams used per day [32,33,35,36,39], years of use and use per week [40], and number of days used over a specific interval [31,37]. To assess cannabis use, five studies used the Structured Clinical Interview for DSM Disorders (SCID) [24,31,33,35,36,38], two used self-report [39,40], and two used a combination of assessments [32,37]. Six of the included studies were conducted in North America [31,32,33,35,36,37,39]. None of our included studies provided follow-up assessments in the weeks or months following neurostimulation, making it difficult to know whether effects persisted following treatment.

### 3.3. Cortical Inhibition and Excitation

Four studies investigated the effects of TMS on cortical inhibition and excitation [24,34,35,38], with three of the studies targeting the motor cortex [24,34,38], and one study targeting the central sulcus [34] (Table 2). Fitzgerald and colleagues compared cortical excitation and inhibition in heavy cannabis users, light cannabis users, and non-using healthy controls [34]. They found reductions in SICI in cannabis users compared to controls with no significant difference between heavy and light cannabis users, and no differences in other cortical excitation and inhibition measures. Similarly, Martin-Rodriguez and colleagues found reduced SICI in cannabis users with and without CUD compared to non-users, with a significant correlation between SICI and plasma THC levels [24]. Wobrock and colleagues conducted a study comparing cortical excitation and inhibition in first-episode schizophrenia patients with and without cannabis abuse and found that patients with cannabis abuse had reduced SICI following right motor cortex stimulation and increased ICF [37]. By contrast, results differed in a study looking at the effects of TMS to the left motor cortex in four groups: cannabis dependence with schizophrenia/schizoaffective disorder, non-cannabis users with schizophrenia/schizoaffective disorder, “non-psychiatric” cannabis dependent participants, and non-psychiatric non-users [37]. Similar to other findings, decreased SICI was observed in individuals who were cannabis dependent compared to non-users, but in contrast to the findings of Wobrock and colleagues, SICI was greater in cannabis dependent patients with schizophrenia compared to patients with schizophrenia who did not use cannabis [34]. This study did not find any differences in LICI between groups. ICF was greater in individuals with schizophrenia, but cannabis use did not affect ICF.

### 3.4. Cannabis Use and Craving Outcomes

Two studies examined the effects of single rTMS sessions on cravings for cannabis (Table 3). A within-subject crossover study randomized participants with CUD to sham or active high-frequency rTMS to the left DLPFC during a cannabis cue paradigm [32]. Although rTMS was well-tolerated, this study found no significant change in total craving scores between active and sham conditions. Another crossover study randomized participants with and without cannabis use to single sessions of high-frequency (10 Hz) and low-frequency (1 Hz) rTMS targeting the posterior cingulate cortex and precuneus [31]. There were no changes in craving after either session.

Two studies investigated the effects of 20 sessions of rTMS on cannabis use [33,36,37]. A case series presented data on nine participants who enrolled in two-weeks of rTMS targeting the left DLPFC for moderate to severe CUD. Patients were also treated with two sessions of weekly motivational enhancement therapy (MET). Of the nine enrolled participants, six dropped out prior to or after treatment initiation and three completed the treatment protocol. Among the three completers, there were reductions in cannabis use and craving scores, although the statistical significance of these changes was not reported [37]. Another study randomized 19 patients with CUD and schizophrenia or schizoaffective disorder to 20 sessions of sham or active rTMS targeting the bilateral DLPFC over a 4-week period [33,36]. There were non-significant reductions in self-reported grams per day of cannabis use in the active group (Cohen’s d = 0.72); an equal proportion of participants abstained from cannabis in both groups [33,36]. There were no significant differences in craving between the active and sham groups. Positive symptom severity decreased in the active group and increased in the sham group as measured by the PANSS, but there were no differences in general or negative symptoms. Treatment retention was high, with 17/19 participants completing treatment (which was facilitated by a contingency management model of participant compensation) with no differences in adverse events between the two arms [33,36].

Finally, two studies examined the effects of tDCS on cannabis craving. Boggio and colleagues randomized cannabis users to right anodal/left cathodal, right anodal/left cathodal, or sham tDCS to the DLPFC for 15 min [40]. The right anodal/left cathodal configuration resulted in a significant decrease in cannabis craving, and none of the participants experienced significant adverse events. By contrast, Patel and colleagues also randomized cannabis users to right anodal/left cathodal or sham stimulation of the DLPFC for 15 min, but found no significant changes in craving in either group [39].

## 4. Discussion

Our results suggest that a single session of rTMS does not alter cravings for cannabis. Delivering rTMS over multiple sessions appears to be more promising. In cannabis users without schizophrenia, a case series found reductions in cannabis use, although the small sample size and the lack of a control group makes it difficult to know whether rTMS was responsible for these changes, especially given that participants were also being treated with therapy. For cannabis users with schizophrenia or schizoaffective disorder, there were reductions in cannabis use with active versus sham rTMS, but these changes did not reach statistical significance. Neither of these studies found significant changes in total cannabis craving scores. rTMS was well-tolerated in all of the studies included in this review. Overall, it seems that a longer course of rTMS may have potential for the treatment of CUD, but data remain sparse and adequately powered studies are needed. Furthermore, none of the included studies provided longer-term follow up after treatment, making it unclear whether the effects of neurostimulation persist.

In terms of rTMS methodology, the most common brain target for studies investigating cannabis craving and consumption was the dorsolateral prefrontal cortex, and the most common coil type was a figure-eight coil. Typically, high frequency (usually 10 Hz) stimulation was used. One study targeted another site (the posterior cingulate cortex and precuneus), but this study did not find any effect on craving. The same study was the only attempt to investigate the effects of low frequency rTMS on craving. Future studies should test other brain targets such as the insula, which has been an effective target for smoking cessation [20,21], and determine whether multiple sessions of low frequency stimulation are effective at reducing cannabis use and craving. Furthermore, use of neuroimaging or neurophysiological biomarkers are being increasingly studied to individualize and increase precision of neuromodulation treatments. Given the anatomical heterogeneity between individuals, the use of individual MRI-guided “neuronavigation” may allow for better localization of target brain regions [41]. It has been demonstrated that even within regions such as the DLPFC, there is variability in response rates depending on the subregion of the DLPFC that is being stimulated [42]. Furthermore, the use of electric field modeling techniques is paving the way for predicting the spread and distribution of the magnetic field in the brain tissue based on the orientation of the coil relative to the spatial distribution of the neurons being stimulated [43]. Lastly, the use of EEG allows for measurement of brain activity with high temporal resolution, potentially allowing for “closed loop stimulation” that delivers stimulation based on the detection of spontaneous or cue-induced symptoms related to the disorder being targeted [44]. In light of the mixed and inconsistent results found in this review, the use of these novel techniques represent promising avenues for improving clinical outcomes.

Studies using TMS to investigate cortical inhibition and excitation have yielded some interesting findings. Three studies found that SICI was decreased in cannabis users compared to non-using controls. This may indicate that chronic cannabis users have decreased activity in cortical inhibitory circuits. SICI is specifically thought to reflect GABA_A_ activity [28], therefore this may indicate that GABA_A_ synapses are altered by chronic cannabis use. In contrast, in individuals with schizophrenia and cannabis use, findings were mixed, with one study showing decreased SICI in individuals with schizophrenia and cannabis abuse and another showing increased SICI in this population. This could reflect differences between the study populations (e.g., differences in positive or negative symptoms or prescribed antipsychotic medications). Future studies will need to address these potential confounds. Other than the SICI findings, there were no other consistently observed differences in cortical inhibition or excitation between cannabis users and controls.

The results from tDCS studies in cannabis users are contradictory. One study found that a single session of right anodal/left cathodal stimulation to the DLPFC decreased cravings for cannabis in regular users, whereas another found no effect of right anodal/left cathodal DLPFC stimulation on cannabis craving. No studies have tested the effects of tDCS on cannabis use or examined the effects of multiple sessions of tDCS on cannabis-related outcomes.

Existing studies of neuromodulation for CUD have important demographic limitations. Firstly, despite the fact that the prevalence of CUD has increased markedly among women [45], three of the studies in this review included no women in at least one study group. In the only two studies that investigated multiple sessions of rTMS for the treatment CUD, only 2 of the 22 participants were women. This makes it difficult to extrapolate whether rTMS could be effective at reducing cannabis consumption in women, especially given important sex-related considerations (e.g., anatomical differences, hormonal differences) that could affect response to rTMS treatment [46] and some evidence from human laboratory studies that women may consume and respond to cannabis differently than men [47]. Trends in high school students suggest that females may be starting to use cannabis at higher rates than males, so it will be increasingly important to balance future studies by gender [48,49]. Another important demographic limitation is that studies primarily recruited participants below the age of 40. Although cannabis use is most common in young adults [50], rates of CUD are increasing in older individuals [45]. The consequences of cannabis use in older individuals may be more severe; for example, cannabis use may be associated with increased risk of ischemic stroke in older individuals [51], and there is a significant impact of cannabis use on driving in this population [52]. It may be worthwhile, once the literature is more developed, to specifically design studies investigating the use of neuromodulation in older individuals with CUD.

At present, there are several ongoing trials testing rTMS and tDCS for heavy cannabis use. A study led by Kearny-Ramos at New York State Psychiatric Institute (NYSPI) is investigating the effects iTBS targeting the DLPFC over the course of ten sessions in two weeks in comparison with sham to evaluate changes in cannabis self-administration in a laboratory setting (NCT05401929). Another study led by Dr. Tang at the Centre for Addiction and Mental Health in Toronto is investigating the feasibility and tolerability of a 4-week course of either high frequency (10 Hz) or low frequency (1 Hz) rTMS targeting the PFC/insula, as well as effects on cannabis use measures (NCT05859347). A study led by Dr. Sahlem at Stanford is investigating change in frequency of daily cannabis use following 36 sessions of either LF rTMS targeting the vmPFC and HF rTMS targeting the DLPFC in combination with 3 sessions of counseling therapy (NCT05720312). Other studies are investigating tDCS treatment for cannabis use with comorbid schizophrenia (NCT05784961, NCT04871048) or multiple sclerosis (NCT05005013). The results of these studies will help determine whether rTMS and tDCS represent viable treatment strategies for CUD, and will provide results that will inform the design of future studies in this field.

## 5. Conclusions

While there is a growing interest in the potential benefits of using brain stimulation to treat problematic cannabis use, our scoping review found that the current body of research on this topic is limited. The scientific literature in this area is still in its early stages, with relatively few studies conducted and a lack of robust evidence to support definitive conclusions. It would be beneficial to further investigate the effects of multiple treatment sessions, as well as to explore whether sex influences treatment effects. It will also be important to investigate combined treatments, such as rTMS combined with psychotherapy interventions. The complex nature of cannabis use disorder, involving both physiological and psychological factors, may eventually require a comprehensive approach that combines brain stimulation techniques with pharmacotherapy, psychotherapy, behavioral interventions, and other support systems.

## Figures and Tables

**Table 1:** Study Characteristics.

Study	Stimulation Type	Study Type	Groups	n (M/F)	Age (Mean [SD])	% Female	Country	Sample Type	Cannabis Use (Mean)	Assessments to Define Groups
Bidzinski et al. (2022) [33]; Johnston et al. (2022) [36]	rTMS	RCT	Active	9 (9/0)	34.78 (3.45)	0	Canada	Cannabis users with CUD and Schizophrenia/Schizoaffective Disorder	Active: 0.77 g/day 10.37 joint-years Sham: 0.85 g/day 6.59 joint-years	SCID
			Sham	10 (9/1)	29.10 (1.70)	10				
Boggio et al. (2010) [40]	tDCS	RCT	Sham	8 (4/4)	22.4 (2.2)	50	Brazil	Cannabis Users Healthy	Sham: 5.1 uses/week 5.9 years of use Right anodal/left cathodal: 5.4 uses/week 5.2 years of use Left anodal/right cathodal: 6.1 uses/week 6.3 years of use	Self-reported at least 3 times per week for 3 years
			Right anodal/left cathodal	9 (5/4)	23.1 (3.5)	44.4				
			Left anodal/right cathodal	8 (6/2)	22.9 (2.0)	25				
			Sham	12 (4/8)	21.3 (2.1)	75				
			Right anodal/left cathodal	12 (3/9)	19.5 (1.0)	75				
			Left anodal/right cathodal	12 (4/8)	20.0 (1.4)	75				
Fitzgerald et al. (2009) [34]	TMS	Cross-sectional	Heavy Users (7+ uses/week)	25 (20/5)	28.56 (9.45)	20	Australia	Cannabis Users Non Users	Not reported	Groups defined by frequency of cannabis use (heavy users = 7 + times per week, light users = 1–4 times per week)
			Light Users (1–4 uses/week)	17 (11/6)	25.12 (6.94)	35.2				
			Non Users	19 (13/6)	28.89 (9.05)	31.6				
Goodman et al. (2016) [35]	TMS	Cross-sectional	Schizophrenia + Cannabis Dependence	12 (12/0)	29.4 (8.4)	0	Canada	Cannabis UsersSchizophrenia	Schizophrenia + CD: 1.2 g/day CD without schizophrenia: 2.0 g/day	SCID
			Schizophrenia Non-User	11 (7/4)	38.5 (8.9)	36.4				
			Cannabis Dependence without Schizophrenia	10 (10/0)	30.4 (7.4)	0				
			Non psychiatric Non User	13 (10/3)	33.5 (10.5)	23.1				
Martin-Rodriguez et al. (2020) [24]	rTMS	Cross-sectional	Non Users	15 (15/0)	25 (3)	0	Spain	Cannabis Users	Not reported	“Based on DSM-5 criteria”
			Cannabis users without CUD	14 (14/0)	24 (3)	0				
			CUD	14 (14/0)	23 (3)	0				
Patel et al. (2022) [39]	tDCS	RCT	Active	15 (8/7)	32.13 (11.19)	46.7	Canada	Cannabis Users	Active: 1.65 g/day Sham: 1.57 g/day	Self-reported at least 3 times per week
			Sham	12 (8/4)	32.92 (11.23)	33.3				
Prashad et al. (2019) [31]	rTMS	Cross-over	Cannabis	10 (5/5)	27.1 (4.5)	50	US	Cannabis Users	Cannabis: 76.7 days of use in preceding 90 days Control: 0 days of use in preceding 90 days	SCID
			Control	10 (5/5)	33.9 (14.1)	50				
Sahlem et al. (2018) [32]	rTMS	Cross-over	Sham then rTMS	7 (6/1)	24.6 (3.7)	14.3	US	Cannabis Users with CUD	Sham first: 1.4 g/day rTMS first: 1.3 g/day	MINI, TLFB, CUDIT
			rTMS then sham	9 (7/2)	27.2 (10.1)	22.2				
Sahlem et al. (2020) [37]	rTMS + MET	Case series	CUD	3 (2/1)	38.3 (11.2)	33.3	US	Cannabis Users with CUD	>20 days of use per month as per inclusion criteria	MINI, TLFB
Wobrock et al. (2010) [38]	TMS	Cross-sectional	Schizophrenia + cannabis abuse	12 (10/2)	24.4 (6.6)	16.7	Germany	Cannabis Abuse with Schizophrenia	Not reported	SCID
			Schizophrenia without cannabis abuse	17 (11/6)	33.6 (7.6)	35.3				

rTMS = repetitive transcranial magnetic stimulation, RCT = randomized clinical trial, CUD = cannabis use disorder, SCID = structured clinical interview for DSM, tDCS = transcranial direct current stimulation, TMS = transcranial magnetic stimulation, MINI = mini-international neuropsychiatric interview, TLFB = timeline follow back, CUDIT = cannabis use disorder identification test.

**Table 2:** Cortical Inhibition and Excitation Outcomes.

Study	Stimulation Type	Groups	n (M/F)	Target Area	Number of Sessions	Intensity	Frequency/Duration	Assessments/Measures	Results
Fitzgerald et al. (2009) [34]	TMS	Heavy Users (7+ uses/week)	25 (20/5)	Central sulcus using 70 mm figure eight coil	1	120 and 140% rMT (CSP) 95% AMT (SICI and ICF) 115 and 130% above rMT (MEP) 120% rMT (LICI)	Controlled single stimulus 2, 3 ms (inhibition) 15 ms (facilitation) 5 s intervals for paired stimuli	SICI, LICI, MEPs, ICF, CSP	Reduced SICI in heavy and light cannabis users compared to non-using controls control at 2 ms ISI and a trend-level difference at 3 ms ISI (*p* = 0.063). No difference in size of CSP at 120% or 140%, MEP size, LICI, or ICF between groups.
		Light Users (1–4 uses/week)	17 (11/6)						
		Non Users	19 (13/6)						
Goodman et al. (2016) [35]	TMS	Schizophrenia + Cannabis Dependence	12 (12/0)	Left motor cortex corresponding to the right abductor pollicis brevis (APB) muscle Figure-eight coil	1	Conditioning stimulus 80% rMT preceded suprathreshold test stimulus set to 1 mv (SICI, ICF) Suprathreshold conditioning and test 1 mV (LICI) 140% rMT (CSP)	One of five interstimulus intervals (ISIs) 2 and 4 ms (SICI) 10, 15, 20 ms (ICF) 100, 150, and 200 ms (LICI)	SICI, LICI, ICF, CSP	SICI greater in cannabis-dependent schizophrenia patients compared to cannabis-free schizophrenia patients. Cannabis-dependent controls had reduced SICI relative to cannabis-free controls. No effects of cannabis dependence on LICI, CSP, and ICF.
		Schizophrenia Non-User	11 (7/4)						
		Cannabis Dependence without Schizophrenia	10 (10/0)						
		Non-psychiatric non-user	13 (10/3)						
Martin-Rodriguez et al. (2020) [24]	rTMS	Non Users	15 (15/0)	Primary motor cortex (Left) with figure-eight coil	2, a week apart	80% AMT	SICI assessed at intensity of 80% AMT with 2 ms interstimulus interval 2 conditions, separated by a week: iTBS, 3 pulses of 50 Hz at 200 ms intervals for 2 s (10 bursts) for a total of 600 pulses cTBS, 40 strain of uninterrupted TBS (600 pulses)	MEPs	At baseline, reduced SICI in cannabis users with and without CUD. Following cTBS, MEPs were inhibited in non-users and cannabis users without CUD, but not in those with CUD. In iTBS, there were no significant differences in MEP inhibition between groups.
		Cannabis users without CUD	14 (14/0)						
		CUD	14 (14/0)						
Wobrock et al. (2010) [38]	TMS	Schizophrenia + cannabis abuse	12 (10/2)	Left and right motor cortex with figure-eight coil	1	Conditioning stimulus 80% rMT (SICI, ICF) 120%, 140%, 160%. 180% rMT (CSP)	3 ms (SICI) 15 ms (ICF)	SICI, ICF, CSP	Reduction of SICI and increase of ICF with right motor cortex stimulation for SZ-SUD group compared to SZ-NSUD group. SICI: (Z = −2.2, df = 1, *p* = 0.026; Mann–Whitney U test). ICF: (Z = −2.0, df = 1, *p* = 0.047; Mann–Whitney U test).
		Schizophrenia without cannabis abuse	17 (11/6)						

TMS = transcranial magnetic stimulation, rMT = resting motor threshold, CSP = cortical silent period, AMT = active motor threshold, ICF = intercortical facilitation, MEP = motor evoked potential, LICI = long intracortical inhibition, SICI = short intercortical inhibition, ISI = interstimulus interval, CUD = cannabis use disorder, iTBS = intermittent theta burst stimulation, cTBS = continuous theta burst stimulation, SZ = schizophrenia, SUD = substance us disorder.

**Table 3:** Cannabis Use and Craving Outcomes.

Study	Stimulation Type	Groups	n (M/F)	Target Area	Number of Sessions	Intensity	Frequency/Duration	Assessments	Results
Bidzinski et al. (2022) [33]; Johnston et al. (2022) [36]	rTMS	Active	9 (9/0)	Left and Right DLPFC (EEG coordinates for F5/F6) Figure-eight coil (MagProX100 B65 Coil)	20 (5×/week over 4 weeks)	90% RMT	20 Hz (25 trains, 30 pulses per train, 30 s intertrain interval)	Cannabis Use (grams per day, Narcocheck) Cannabis craving (MCQ) Cognitive Performance Neurocognitive battery Verbal memory, visuospatial working memory, sustained attention, delay discounting, complex planning	No significant differences in cannabis use between groups, although reductions in grams per day of cannabis use were greater in the active vs. the sham group. Total MCQ score was not significantly different between groups. PANSS positive scores decreased in severity in the active group and increased in severity in the sham group, although there was no significant treatment by time interaction.
		Sham	10 (9/1)						
Boggio et al. (2010) [40]	tDCS	Sham	8 (4/4)	DLPFC Left anodal/right cathodal (Anode over left F3, cathode over right F4) Right anodal/left cathodal (Anode over right F4, cathode over left F3)	1	2 mA intensity with 10 s of ramp up and down	15 min (5 min before risk task, 10 min for duration of risk task)/Sham group received only 30 s of stimulation.	Marijuana craving before and after stimulation (Visual analog scale) Risk Task	Right anodal/left tDCS resulted in a significant decrease in marijuana craving whereas as there was no significant change in the left anodal/right cathodal configuration or sham condition
		Right anodal/left cathodal	9 (5/4)						
		Left anodal/right cathodal	8 (6/2)						
		Sham	12 (4/8)						
		Right anodal/left cathodal	12 (3/9)						
		Left anodal/right cathodal	12 (4/8)						
Patel et al. (2022) [39]	tDCS	Active	15 (8/7)	DLPFC Right anodal/left cathodal (Anode over right F3, cathode over left F4)	1	Active: 2 mA for 15 min, ramp up time of 30 s Sham: Received ramp up but current stopped after 30 s	15 min	Cannabis Craving Risk Tasking (Gambling Task) Delay Discounting and Probability Discounting	No significant difference in craving
		Sham	12 (8/4)						
Prashad et al. (2019) [31]	rTMS	Cannabis	10 (5/5)	Posterior cingulate cortex and precuneus Double-cone coil	1 low frequency, 1 high frequency [Cross-over design: low frequency (1 Hz) and high frequency (10 Hz) sessions 1 week apart]	80% of resting motor threshold (rMT) or 45% of maximum stimulator output (MSO) rMT exceeded 45% MSO Lower of the two	LF 1 Hz HF 10 Hz 2000 pulses	MCQ	No significant change in MCQ scores following low frequency or high frequency rTMS
		Control	10 (5/5)						
Sahlem et al. (2018) [32]	rTMS	Sham then rTMS	7 (6/1)	Left DLPFC (EEG Coordinate for F3 used) Figure-eight coil	Cross-over design: 1 Active, 1 Sham	110% of resting motor threshold (rMT)	4000 pulses at 10 Hz 5 s on, 10 s off, delivered during a cannabis cue paradigm	Cravings (MCQ) 1. Baseline 2. Following neutral cue during rTMS 3. Following active cue during rTMS 4. Immediately following rTMS 5. 15 min after rTMS completion	Retention rate was 89% (14/16 participants retained) and there were no treatment related complications. No difference in MCQ total score between active and sham conditions during or following rTMS.
		rTMS then sham	9 (7/2)						
Sahlem et al. (2020) [37]	rTMS	Active	3 (2/1) Treatment completers (9 enrolled, 6 dropped out)	Left DLPFC (EEG Coordinate for F3 used) Figure-eight coil	20, over the course of 2 weeks (10 treatment visits with 2 rTMS sessions per visit) Patients also received 2 sessions of weekly MET	120% of rMT	4000 pulses at 10 Hz 5 s on, 10 s off delivered during a cannabis cue paradigm	Cravings (MCQ) at baseline and after each session Cannabis Use using TLFB	Mean MCQ-SF scores decreased by approximately 16 points, from 50.3 ± 7.1 prior to treatment to 34.0 ± 26.2 prior to the final visit (Cohen’s d = 1.3) Cannabis use decreased from 31.7 ± 15.1 use-sessions per week at baseline to 17.0 ± 13.5 SD in the week following treatment. One participant dropped out due to headaches.

rTMS = repetitive transcranial magnetic stimulation, DLPFC = dorsalateral prefrontal cortex, RMT = resting motor threshold, MCQ = marijuana craving questionnaire, tDCS = transcranial direct current stimulation, MET = motivation enhancement therapy, TLFB = timeline followback.

## Data Availability

Data from this study can be made available upon request. The data are not publicly available due to privacy reasons.

## Supplementary Materials

The following supporting information can be downloaded at: https://www.mdpi.com/article/10.3390/brainsci14040356/s1. Table S1: PubMed Search Strategy. Table S2: OVID Medline Search Strategy. Table S3: PsycInfo Search Strategy.
