# Supplementary material for: Neuromodulation for Cannabis Use: A Scoping Review
Source: Brain Sci. 2024 Apr 2;14(4):356. doi: 10.3390/brainsci14040356 (PMC11048669; doi:10.3390/brainsci14040356)
Supplement: Supplementary file 1 [file brainsci-14-00356-s001.zip › brainsci-2888016-supplementary.pdf]

Supplementary Table S1: PubMed search strategy

Search #	Query	Items Found
1	“cannabis use dis*” or “cannabis add*” or “cannabis use*” or “cannabis abuse*”	10,578
2	“marijuana add*” or "Marijuana Abuse"[Mesh] or “marijuana use*”	12,596
3	1 OR 2	19,491
4	“brain stimulation” or “neuromodulation” or “neurostimulation”	43,693
5	“Deep Brain Stimulation”[MeSH] or “DBS” or “Deep Brain Stimulation”	22,034
6	“Transcranial Magnetic Stimulation”[MeSH] or “Vagus Nerve Stimulation”[MeSH] or “TMS” or “rTMS” or “TBS” or “VNS” or “Transcranial magnetic stimulation” or “Repetitive transcranial magnetic stimulation” or “Theta Burst Stimulation”	36,104
7	“Electroconvulsive Therapy”[MeSH] or “Transcranial Direct Current Stimulation”[MeSH] or “ECT” or “tDCS” or “Electroconvulsive therapy” or “Transcranial Direct Current Stimulation”	28,327
8	“Electric Stimulation Therapy”[MeSH] or “Nerve Stimulation”	110,878
9	“Extracorporeal Shockwave Therapy”[MeSH] or “Focused Ultrasound”	9,214
10	4 OR 5 OR 6 OR 7 OR 8 OR 9	194,510
11	3 AND 10	49

Supplementary Table S2: OVID Medline search strategy

Search #	Query	Items Found
1	exp Cannabis/ or cannabis*.mp. or cannabinoids*.mp or exp Cannabinoids/	45,710
2	marijuana*.mp. or exp Marijuana Smoking/ or exp Marijuana Abuse/ or exp "Marijuana Use"/	24,676
3	1 OR 2	56,270
4	(Neuromodulation adj1 (intervention* or treatment* or therap*)).mp. or brain stimulation*.mp.	24,330
5	exp Deep Brain Stimulation/ or DBS.mp. or Deep Brain Stimulation.mp	21,097
6	exp Transcranial Magnetic Stimulation/ or rtms.mp. or Transcranial Magnetic Stimulation.mp	21,682
7	tdcs.mp. or exp Transcranial Direct Current Stimulation/ or Transcranial Magnetic Stimulation.mp	27,517
8	exp Electroconvulsive Therapy/ or ect.mp. or Electroconvulsive Therapy.mp	20,099
9	exp Vagus Nerve Stimulation/ or exp Electric Stimulation/ or nerve stimulation.mp. or exp Electric Stimulation Therapy/	230,072
10	focused ultrasound.mp. or exp Ultrasonic Therapy/	18,091
11	4 or 5 or 6 or 7 or 8 or 9 or 10	295,549
12	3 AND 11	481

Supplementary Table S3: Psychinfo search strategy

Search #	Query	Items Found
1	exp "Cannabis Use Disorder"/ or exp Cannabis/ or cannabis*.mp. or cannabinoids.mp. or exp Cannabinoids/	25,055
2	exp Marijuana/ or exp Marijuana Usage/ or marijuana*.mp.	16,689
3	1 OR 2	30,729
4	exp Neuromodulation/ or neuromodulation*.mp. or exp Brain Stimulation/ or brain stimulation*.mp.	31,065
5	exp Deep Brain Stimulation/ or dbs.mp. or Deep brain stimulation.mp	6090
6	exp Transcranial Magnetic Stimulation/ or tms.mp. or Transcranial Magnetic Stimulation.mp	13,459
7	exp Transcranial Direct Current Stimulation/ or tdcs.mp or Transcranial Direct Current Stimulation.mp	4392
8	exp Electroconvulsive Shock Therapy/ or ect.mp. or Electroconvulsive Therapy.mp	11,417
9	exp Vagus Nerve/ or exp Nerve Stimulation/ or exp Electrical Stimulation/ or nerve stimulation*.mp.	29,248
10	exp Ultrasound/ or focused ultrasound.mp.	2031
11	4 or 5 or 6 or 7 or 8 or 9 or 10	54,795
12	3 AND 11	202
